# Identification of QTLs and candidate genes for water-soluble protein content in soybean seeds

**DOI:** 10.1186/s12864-024-10563-0

**Published:** 2024-08-13

**Authors:** Xujuan Zhang, Fengmin Wang, Qiang Chen, Qingsong Zhao, Tiantian Zhao, Xuejie Hu, Luping Liu, Jin Qi, Yake Qiao, Mengchen Zhang, Chunyan Yang, Jun Qin

**Affiliations:** 1 Hebei Laboratory of Crop Genetics and Breeding, Huang-Huai-Hai Key Laboratory of Biology and Genetic Improvement of Soybean, Ministry of Agriculture and Rural Affairs, Institute of Cereal and Oil Crops, National Soybean Improvement Center Shijiazhuang Sub-Center, Hebei Academy of Agricultural and Forestry Sciences, Shijiazhuang, Hebei China; 2 College of Agronomy and Biotechnology, Hebei Normal University of Science and Technology, Qinhuangdao, China; 3 Hebei Key Laboratory of Molecular and Cellular Biology, Key Laboratory of Molecular and Cellular Biology of Ministry of Education, Hebei Collaboration Innovation Center for Cell Signaling, College of Life Science, Hebei Normal University, Shijiazhuang, China

**Keywords:** Soybean, WSP, QTL, Candidate gene mining

## Abstract

Soybean represents a vital source of premium plant-based proteins for human nutrition. Importantly, the level of water-soluble protein (WSP) is crucial for determining the overall quality and nutritional value of such crops. Enhancing WSP levels in soybean plants is a high-priority goal in crop improvement. This study aimed to elucidate the genetic basis of WSP content in soybean seeds by identifying quantitative trait loci (QTLs) and set the foundation for subsequent gene cloning and functional analysis. Using 180 F_10_ recombinant inbred lines generated by crossing the high-protein soybean cultivar JiDou 12 with the wild variety Ye 9, our researcher team mapped the QTLs influencing protein levels, integrating Kyoto Encyclopedia of Genes and Genomes (KEGG) pathway analysis and gene expression profiling to identify candidate genes. During the 2020 and 2022 growing seasons, a standard bell-shaped distribution of protein content trait data was observed in these soybean lines. Eight QTLs affecting protein content were found across eight chromosomes, with LOD scores ranging from 2.59 to 7.30, explaining 4.15–11.74% of the phenotypic variance. Notably, two QTLs were newly discovered, one with a elite allele at *qWSPC-15* from Ye 9. The major QTL, *qWSPC-19*, on chromosome 19 was stable across conditions and contained genes involved in nitrogen metabolism, amino acid biosynthesis, and signaling. Two genes from this QTL, *Glyma.19G185700* and *Glyma.19G186000*, exhibited distinct expression patterns at maturity, highlighting the influence of these genes on protein content. This research revealed eight QTLs for WSP content in soybean seeds and proposed a gene for the key QTL *qWSPC-19*, laying groundwork for gene isolation and enhanced soybean breeding through the use of molecular markers. These insights are instrumental for developing protein-rich soybean cultivars.

**Supplementary Information:**

The online version contains supplementary material available at 10.1186/s12864-024-10563-0.

## Introduction

Soybean is an important leguminous plant with seeds that are rich in protein and that serve as a crucial source of high-quality plant protein for humans [1]. Water-soluble protein (WSP) is a significant component of soybean seed protein and not only play a key role in the quality of soy food products and the yield of protein isolates [2] but also play important roles in gelation, emulsification, and foaming in food processing and utilization [3]. Thus, enhancing the WSP content in soybean seeds has significant implications for soybean quality and food processing.

Cultivated soybeans were domesticated more than 5000 years ago [4, 5], and throughout their lengthy domestication process, they acquired physiological, morphological, and genetic traits that differed from those of wild soybeans, with these phenotypic differences fundamentally arising from variations in relevant functional genes [6]. The protein content of wild soybean plant seeds is greater than that of current cultivated varieties, suggesting the presence of genes controlling high protein content in these wild plants [7, 8]. However, there is a lack of research on WSP-related traits in wild soybeans. The WSP content in soybeans is a quantitative trait controlled by multiple genes [9, 10], and to date, there has been little research on the mapping of quantitative trait loci (QTLs) for soybean WSP content, with existing research mainly focused on cultivated varieties. Lu and colleagues [11] used a set of 212 F_2:9_ recombinant inbred lines (RILs) derived from the cross ZDD09454×Yu Dou 12 to locate 11 QTLs related to WSP content. Zhang and others [12] identified 16 significant loci related to soybean WSP content using 219 soybean accession materials and 152 RIL materials through genome-wide association study (GWAS) analysis, among which the major locus GqWSPC8 was consistently identified in multiple environments. Zhang et al. [13]. used 211 soybean accessions to construct a natural population and located 5 QTLs related to WSP content through GWAS. Chen et al. [14]. used a population of 188 RILs developed from a cross between Ji Dou 12 and a semiwild black soybean accession to map 23 QTLs related to WSP content via the bulked segregant analysis method. Although QTLs related to soybean WSP continue to be reported, there has been no research on the genetic background traits related to WSP content in wild soybeans, the high-protein ancestors of domesticated soybeans. This study utilized a set of 180 F_10_ RILs derived from a cross between the cultivated variety Ji Dou 12 and the wild soybean variety Ye 9 as the experimental material. Based on a high-density genetic map, this study aimed to identify QTLs related to WSP content in various environments to reveal the genetic structure underlying soybean WSP. The goal of this study was to identify major and stably inherited QTLs for soybean WSP and to predict candidate genes, providing a theoretical basis for molecular marker-assisted selection breeding and the improvement of soybean quality.

Despite the increasing number of reports on QTLs related to soybean WSP, there is still a lack of in-depth research investigating the genetic background of wild soybean, which is the ancestral source of high protein quality in soybeans. Considering that the ancestors of domesticated soybeans are wild soybean plants with high protein traits, exploring the genetic mechanisms underlying WSP content is both important and of high priority.

This study aimed to analyze the genetic basis of WSP content within an F_10_ recombinant inbred line (RIL) population derived from a cross between the cultivated soybean variety Ji Dou 12 and the wild soybean variety Ye 9. By detecting and analyzing WSPC-related QTL loci based on a high-density genetic linkage map under multiple environmental conditions, this study sought to elucidate the genetic architecture of WSP in soybeans and aimed to locate pivotal and stably inherited QTLs related to WSP content, along with the preliminary prediction of candidate genes. Our research provides a theoretical basis for marker-assisted selection and supports the improvement and quality enhancement of soybean cultivars.

## Materials and methods

### Experimental materials and field management

The cultivated soybean variety JD12 (ZDD23040, developed by the Institute of Cereal and Oil Crops, Hebei Academy of Agricultural and Forestry Sciences) was crossed with the wild soybean variety Y9 (ZYD02739, collected from Chengde City, Hebei Province, provided by research professor Lijuan Qiu from Institute of Crop Sciences, Chinese Academy of Agricultural Sciences). Using the single-seed descent (SSD) method, a reference population consisting of 185 F8-derived recombinant inbred lines (RILs) was constructed [15]. In the 2020 and 2022 soybean growing seasons, the parents and RIL populations were sown at the Gaochengdi Experimental Station in Shijiazhuang, Hebei Province.Both parents are stored in the the National Crop Genebank(see Supplementary Table 1). Each soybean line was planted in three rows, and each row was 3 m long, with a row spacing of 0.5 m and a plant spacing of 0.2 m. A completely randomized block design was adopted with three repetitions. Normal field management was carried out until the plants naturally matured, after which seeds from five plants in the central row were collected to determine the water soluble protein content. Additionally, 30 wild soybean germplasms from Hebei Province and 30 cultivated soybean varieties from the Huang-Huai-Hai region (see Supplementary Table 2) were randomly selected to test the water soluble protein content.

### Method for determining soybean seed water soluble protein content

The extraction and determination of soybean seed water soluble protein content were carried out according to the methods of Zhang Mengchen et al. [16]. with optimizations made for this experiment.

#### Preparation of the standard curve

Bovine serum albumin standard solution was prepared with double distilled water at different concentrations (0, 0.2, 0.4, 0.6, 0.8, and 1.0 mg·mL^-1^) to serve as working solutions. For each concentration, three technical replicates were used to determine the water soluble protein content. Finally, the standard curve was drawn using Excel.

#### Extraction of water-soluble protein from soybean seeds

The soybeans were fully ground by a grinding machine and then sieved through an 80-mesh screen. A total of 0.020 g of the powder sample (accurate to 0.001 g) was dissolved in 2 mL of distilled water in a centrifuge tube and incubated with constant shaking at 20 °C for 60 min to ensure full dissolution of the water soluble protein. After the centrifuge tube was removed, it was centrifuged at 3000 r/min for 10 min. The supernatant was poured into a 10 mL volumetric flask, and the residue was extracted repeatedly. The residue was then incubated on a shaker at a constant temperature (22 °C) for 30 min, followed by centrifugation at 3500 r/min for 10 min. After combining the extract solutions, the mixture was brought up to a volume of 10 mL with distilled water, creating a sample solution of WSP for testing.

#### Determination of soybean water soluble protein content

Forty microlitres of the sample solution was placed in a centrifuge tube, to which 2 ml of Coomassie Brilliant Blue G-250 dye solution was added. After mixing, the solution was immediately placed into a cuvette, and the absorbance at 595 nm was measured using a spectrophotometer. The absorbance values were subsequently applied to the standard curve equation to calculate the water soluble protein content. The mean of three replicate measurements for each sample was taken as the water soluble protein content of that sample. Phenotypic data were analyzed for descriptive statistics and correlations using SPSS 26.0 software. ANOVA and heritability calculations were performed on the phenotypic data using SAS9.2 software, with the equation for broad-sense heritability as follows:

$${h}^{2}=\frac{{\sigma }_{g}^{2}}{({\sigma }_{g}^{2}+\frac{{\sigma }_{ge}^{2}}{n}+\frac{{\sigma }_{e}^{2}}{rn})}$$

[17] 

where ${\sigma }_{g}^{2}$ represents the genetic variance, ${\sigma }_{ge}^{2}$ represents the genotype × environment interaction variance, ${\sigma }_{e}^{2}$ represents the error term, n is the number of environments, and r is the number of replications.

### Construction of a genetic linkage map and QTL mapping

In this study, the high-density genetic linkage map constructed by Yang et al. [18] was referenced to construct a genetic map totaling 6626.06 cM in length through 3659 SNP molecular markers. The average genetic map length of each chromosome was 331.30 cM, with an average of 183 markers per chromosome and an average genetic distance of 1.81 cM between markers. Analyses were carried out using SMA, IM-ADD, and ICIM-ADD in IciMapping software (V4.2.53), with the additive effect QTL detection threshold set to LOD = 2.5. A QTL was considered to exist if the LOD exceeded this threshold [19].

Epistatic QTL mapping (ICIM-EPI) was conducted using phenotypic values, with the threshold for epistatic interaction effects set at 5.0, and the QTLs were named following the methods of McCouch et al. [20].

### Prediction and analysis of candidate gene expression

Candidate genes related to soybean WSP content were predicted based on gene function annotations within the Phytozome database (https://phytozome.jgi.doe.gov/) and Soybase database (https://www.soybase.org), and metabolic pathway enrichment analysis was performed using the KEGG signaling pathway database.

Among them, N represents the number of genes with KEGG annotations, m represents the number of all genes annotated in a specific pathway, M represents the number of candidate genes in M, and n represents the number of candidate genes in N.

$$p = 1 - \sum\limits_{i - 0}^{m - 1} {\frac{{\left( {\frac{M}{i}} \right)\left( {\frac{{N - M}}{{N - i}}} \right)}}{{\left( {\frac{N}{n}} \right)}}}$$

Genes with known functional descriptions related to soybean protein content or participating in seed protein synthesis pathway were selected as candidate genes.Seeds of the parental lines Ji Dou 12 (JD 12) and Ye 9 were collected at the maturity stage (R6) and immediately frozen in liquid nitrogen for RNA extraction. Total RNA was extracted from the seeds using an RNeasy Plant Mini Kit (OMEGA, USA). Subsequently, 1000 ng of RNA was reverse transcribed to obtain cDNA using TransScript One-Step gDNA Removal and cDNA Synthesis SuperMix (Vazyme, Nanjing, China). Real-time quantitative PCR (qRT–PCR) was performed using the TransScript One-Step gDNA Removal and cDNA Synthesis SuperMix Kit and the Bio-Rad CFX96™ system (Bio-Rad Laboratories, CA, USA) for detection. The volume of each reaction was 20 µL, and the reaction program included predenaturation at 95 °C for 2 min, denaturation at 95 °C for 30 s, and annealing at 60 °C for 30 s for 39 cycles, followed by a final extension at 72 °C for 2 min. The soybean reference gene β-actin was used to normalize expression levels, and the 2^-△△Ct^ method was used to calculate the relative expression levels of each gene [21]. Each qRT‒PCR included 3 technical replicates and 3 biological replicates (for primer sequences, see Supplementary Table 3).

## Results and analysis

### Statistical analysis of water-soluble protein content in germplasm resources and RIL populations

**Fig. 1:**
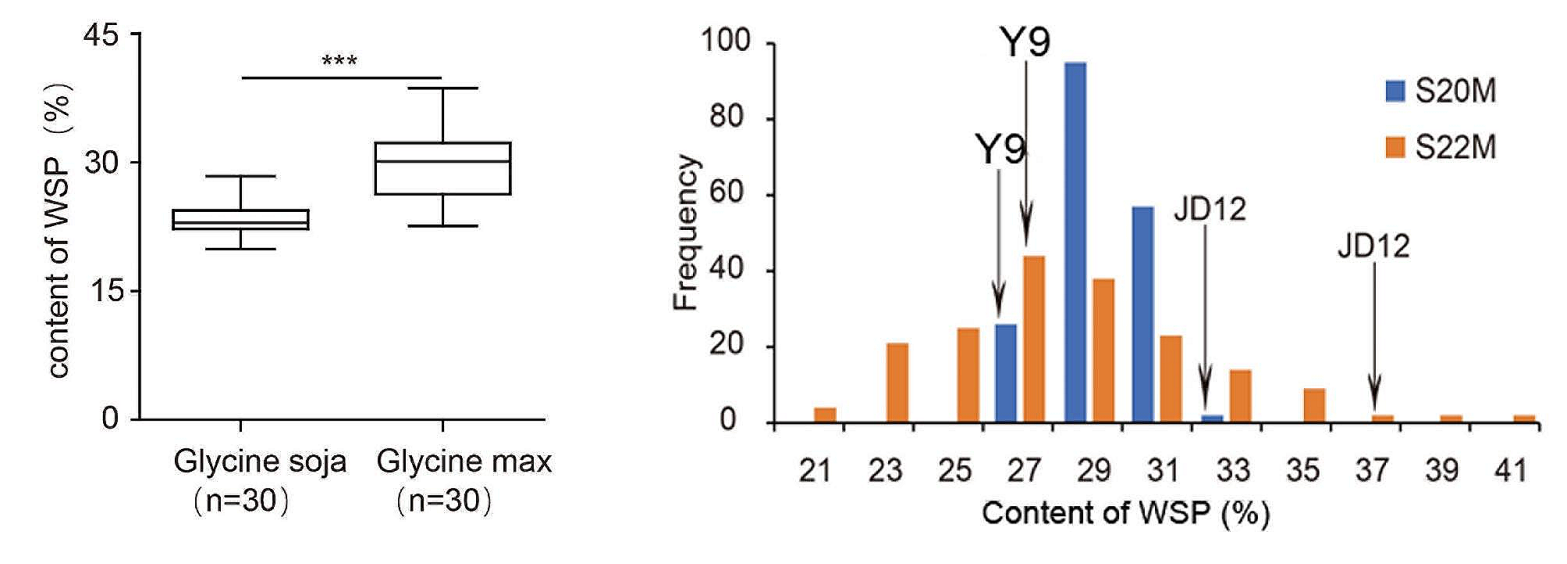
Water-soluble protein content of soybean in germplasm resources and RILs populations. **a** Water-soluble protein content histogram of Glycine soja and Glycine max. **b** Water-soluble protein content histogram of RIL population; JD12 and Y9 represent the water-soluble protein content values of parent Jidou 12 and wild 9, respectively

To explore the patterns of WSP content in wild and cultivated soybeans, 30 samples from each of the two groups were randomly selected for detection of the seed WSP content. The results showed that, in the 30 wild soybean samples, the WSP content ranged between 19.90% and 28.38%, with an average value of 23.42%; in the 30 randomly selected cultivated soybean samples, it ranged between 22.54% and 38.62%, with an average value of 29.29%. The significance analysis revealed that the WSP content in the cultivated varieties was significantly greater than that in the wild soybean plants (Fig. 1A).

To investigate the genetic basis of the high WSP content in cultivated soybeans and to discover superior allelic variations related to WSP in wild soybeans, we used a recombinant inbred line (RIL) population constructed from cultivated soybean Ji Dou 12 and wild soybeans, which differ greatly in WSP content and genetic background. The WSP content in seeds was determined in 2020 and 2022, and the results revealed that the phenotypic distribution of the population tended to be normal and showed transgressive segregation, consistent with the genetic characteristics of quantitative traits and suitable for further QTL mapping analysis (Fig. 1B). A statistical analysis of the WSP content revealed that in these two years, the Ji Dou 12 WSP content was 32.53% and 35.9%, while that of Ye 9 was 26.83% and 25.58%, respectively. The phenotypic range of the RIL population in 2020 was 23.47–32.53%, with an average of 29.29%; in 2022, it was 21.21–42.43%, with an average of 29.1% (Table 1), indicating a broader phenotypic distribution in the 2022 population. The broad-sense heritability for WSP was 57.56%, suggesting that the phenotypic variation was strongly influenced by genetic factors. However, the variance analysis results indicated that the environment and the genotype-environment interaction also had significant impacts on the WSP content.

**Table 1:** Statistical analysis of water-soluble protein content in soybean

Years	Parents		RIL population						Significance			h^2^/%
	JD12	Y9	Rang	SD	CV%	Mean	Skewness	Kurtosis	G	E	G×E	
2020Y	32.53 ± 0.44	26.83 ± 0.10	23.47–32.53	1.2	4%	29.29	-0.936	0.984	**	**	**	57.56
2022Y	35.90 ± 0.91	25.58 ± 0.60	21.21–42.43	3.86	13%	29.1	0.711	0.832				

### QTL mapping for soybean water-soluble protein

**Fig. 2:**
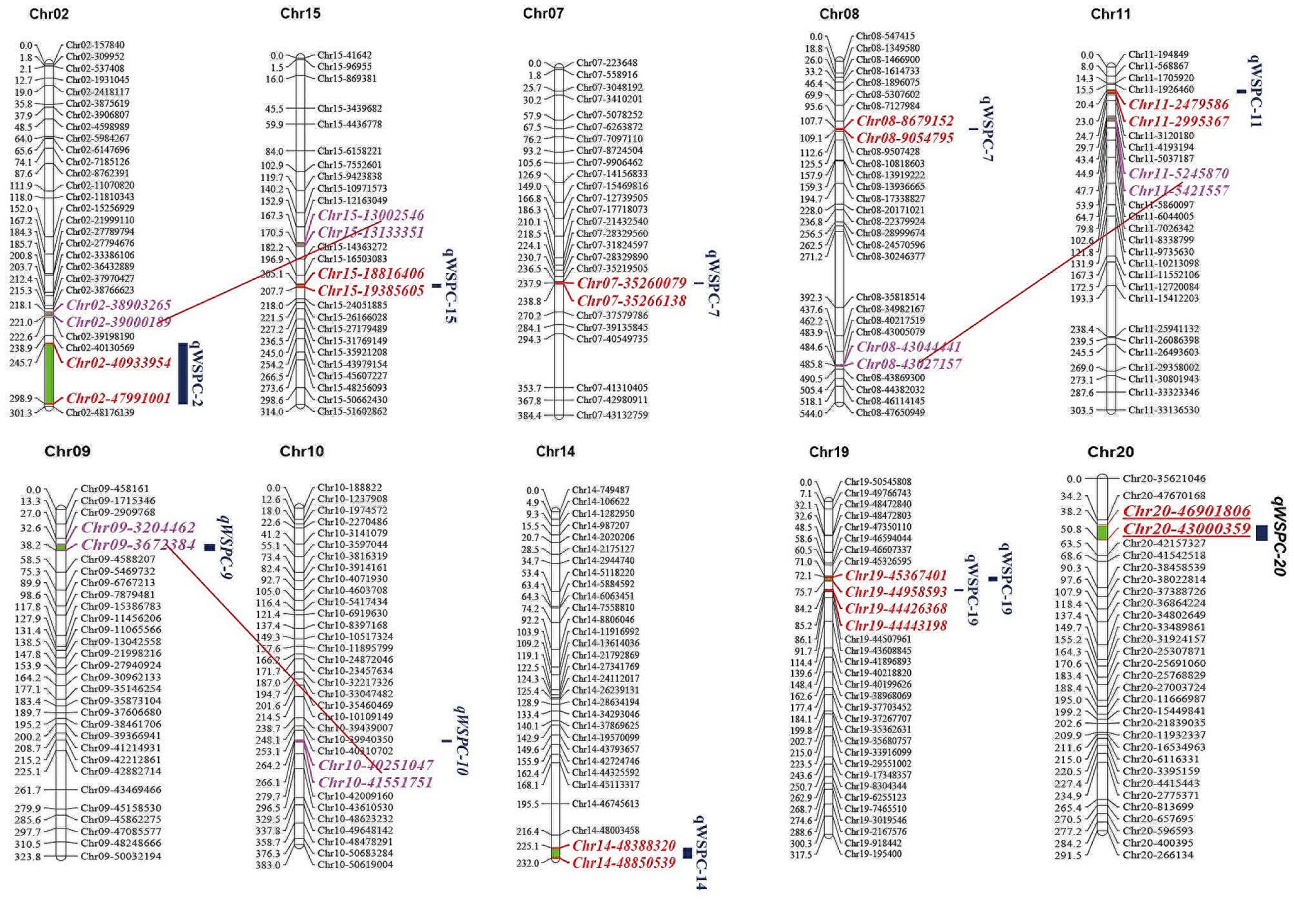
Distribution of additive QTLs and epistatic QTLs on linkage groups in RIL population. the dashed line represents the epistatic interaction between the linked QTLs

Eight QTLs related to soybean WSP content were mapped for the years 2020 and 2022; these QTLs were located on chromosomes 2, 7, 8, 11, 14, 15, 19, and 20 (Table 2; Fig. 2). Four QTLs were mapped in 2020 with LODs ranging from 2.59 to 7.30. These QTLs accounted for between 5% and 11.74% of the phenotypic variation and were distributed on chromosomes 2, 8, 11, and 19, and were named qWSPC-2, qWSPC-8, qWSPC-11, and qWSPC-19, respectively. qWSPC-2 had the highest phenotypic contribution rate and was positioned between the markers Chr02-40933954 and Chr02-47991001, with a LOD value of 2.59 and a phenotypic contribution rate of 11.74%. The additive effect came from the parent Ji Dou 12. The LODs for qWSPC-8, qWSPC-11, and qWSPC-19 ranged from 3.36 to 7.30, with phenotypic contribution rates between 5% and 11.74%.

**Table 2:** Additive QTLs mapping of water-soluble protein content in RIL population

TraitName	Environment	Chromosome	Position	LeftMarker	RightMarker	LOD	PVE(%)	Add	Reference
*qWSPC-2*	S20M	2	263	*Chr02-40933954*	*Chr02-47991001*	2.59	11.74	0.64	Chr02-46353731 [14]
*qWSPC-7*	S22M	7	238	*Chr07-35260079*	*Chr07-35266138*	3.57	4.90	0.96	
*qWSPC-8*	S20M	8	109	*Chr08-8679152*	*Chr08-9054795*	7.30	11.45	0.64	Chr08_8643359 [12]
*qWSPC-11*	S20M	11	23	*Chr11-2479586*	*Chr11-2995367*	3.36	5.00	0.43	Chr11_2674088 [31]
*qWSPC-14*	S22M	14	232	*Chr14-48388320*	*Chr14-48850539*	3.05	4.15	0.88	
*qWSPC-15*	S22M	15	207	*Chr15-18816406*	*Chr15-19385605*	4.97	7.19	-1.17	Chr15-22885468 [14] Chr15-22969031 [32]
*qWSPC-19*	S20M	19	85	*Chr19-44426368*	*Chr19-44443198*	6.22	9.71	0.58	Chr19-42262311 [2]
	S22M	19	74	*Chr19-44958593*	*Chr19-45367401*	5.25	7.75	1.21	Chr19-47615818 [12]
*qWSPC-20*	S22M	20	41	*Chr20-43000359*	*Chr20-46901806*	6.64	11.17	1.46	chr20_40499700 [12]

In the 2022 environment, five QTLs were mapped on chromosomes 7, 14, 15, 19, and 20, with LODs ranging from 3.05 to 6.64 and phenotypic variance explained from 4.15 to 11.17%. These were named qWSPC-7, qWSPC-14, qWSPC-15, qWSPC-19, and qWSPC-20. Among them, qWSPC-20 had the highest phenotypic contribution rate, located between the markers Chr20-43000359 and Chr20-46901806, with an LOD of 6.64 and a phenotypic contribution rate of 11.17%, with the increasing effect allele coming from the parent Ji Dou 12. The increasing effect allele for qWSPC-15 came from the male parent wild soybean Ye 9, with an LOD of 4.97 and a contribution rate of 7.19%. Additionally, the LODs for the other three QTLs, qWSPC-7, qWSPC-14, and qWSPC-19, were 3.57, 3.05, and 5.25, with contribution rates of 4.90%, 4.15%, and 7.75%, respectively. qWSPC-19 was consistently detected in the environments of both years and was positioned between the markers Chr19-44426368 and Chr19-45367401, with the increasing effect allele sourced from Ji Dou 12.

### Detection of epistatic Interaction QTLs for soybean water-soluble protein

Epistatic interaction (ICIM-EPI) QTL mapping was performed using the phenotypic values of the WSP content from the two years. For the year 2020, three sets of QTLs with interaction effects were identified. An interaction was found between chromosome 8 (43,027,157–43,044,441 bp) and chromosome 11 (5,245,870–5,421,557 bp), with an LOD value of 5.43 and a genetic contribution rate of 1.82%. Interactions were observed between chromosome 2 (38,903,265–39,000,189 bp) and chromosome 15 (13,002,546–13,133,351 bp), with an LOD of 5.59 and a contribution rate of 2.15%. For the year 2022, a set of epistatic interaction effect QTLs was located between chromosome 9 (3,204,462–3,672,384 bp) and chromosome 10 (40,251,047–41,551,751 bp), with an LOD value of 5.94 and a genetic contribution rate of 17.66% (Fig. 2). The interactions detected between chromosomes 2 and 15 as well as between chromosomes 8 and 11 were among the additive effect QTLs, suggesting that in addition to additive effects, epistatic interactions play a significant role in the genetic basis of WSP content.

### Candidate gene prediction and validation

**Fig. 3:**
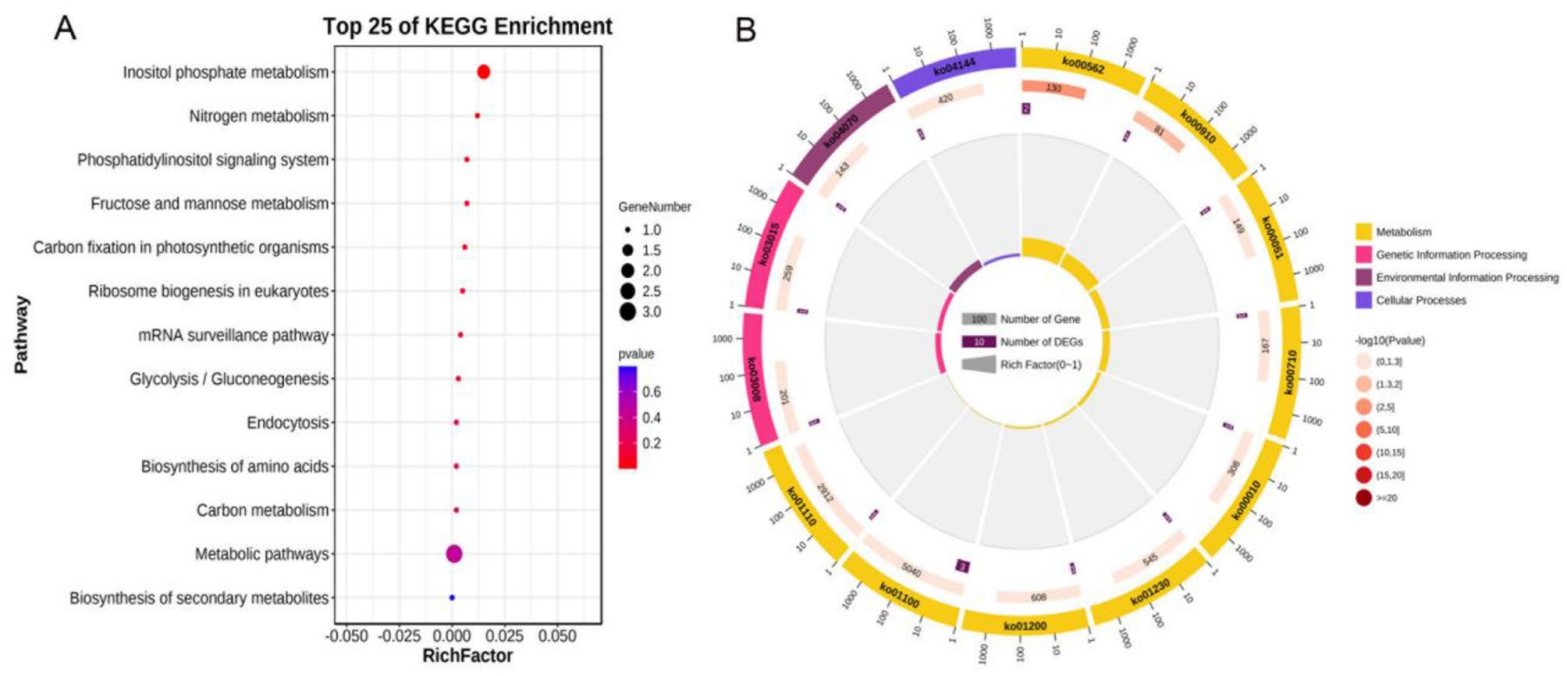
**A.** KEGG analysis of *qWSPC-19* positioning interval genes

**Fig. 4:**
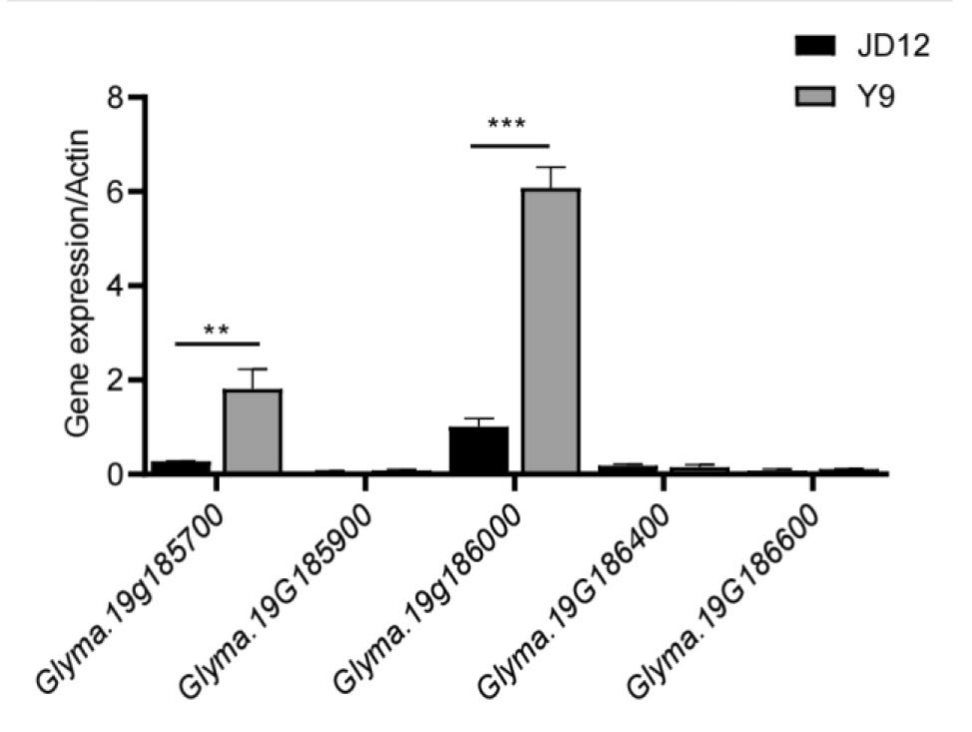
Comparison of the expression levels of two parents by qRT − PCR analysis.Note: **. Significant different at the *P* < 0.01 level; ***. Significant different at the *P* < 0.001 level

*qWSPC-19* is a stable and major-effect quantitative trait locus (QTL) associated with WSP content. With reference to the Phytozome and SoyBase databases, the marker interval of *qWSPC-19* was determined to contain 111 predicted genes. Integrated with the Kyoto Encyclopedia of Genes and Genomes (KEGG) pathway analysis, these genes are primarily enriched in pathways such as inositol phosphate metabolism, nitrogen metabolism, phosphatidylinositol signaling system, fructose and mannose metabolism, carbon fixation in photosynthetic organisms, ribosome biogenesis in eukaryotes, mRNA surveillance pathway, glycolysis/gluconeogenesis, Biosynthesis of amino acids, carbon metabolism, metabolic pathways, and biosynthesis of secondary metabolites (Fig. 3). Five of these genes were found to be involved in biological processes such as nitrogen metabolism, amino acid synthesis, and the phosphatidylinositol signaling system, which are presumed to be related to the WSP content in the region of *qWSPC-19* (Table 3). Transcription level analysis at different developmental stages of seeds from the parents Ye 9 and Ji Dou 12 revealed that two genes, *Glyma.19G185700* and *Glyma.19G186000*, were highly expressed at various stages of seed development, suggesting a potential role in this process. During the later stage of seed development (R6 stage), when the soluble protein content in soybeans reaches its highest level [22], *Glyma.19G185700* and *Glyma.19G186000* showed significantly different expression levels between the two parents, with expression being significantly greater in Ye 9 than in Ji Dou 12 (Fig. 4). Therefore, *Glyma.19G185700* and *Glyma.19G186000* are posited as candidate genes for *qWSPC19* and are likely involved in the synthesis of soybean WSP.

**Table 3:** Candidate genes and functional annotation of soybean water-soluble protein content

QTL	Chromomose	Candidate interval		Candidate gene	Function annotation	Top Arabidopsis (TAIR10) BLASTP Hit	GO Biological Process Descriptions
		Start/bp	End/bp				
*qWSPC-19*	19	44,426,368	45,367,401	*Glyma.19G185700*	mRNA surveillance pathway	AT3G09880.1 Symbols: Protein phosphatase 2 A regulatory B subunit family protein	protein dephosphorylation; megasporogenesis; microsporogenesis; regulation of protein autophosphorylation;
				*Glyma.19G185900*	Nitrogen metabolism	AT1G08080.1 Symbols: alpha carbonic anhydrase 7	
				*Glyma.19G186000*	Biosynthesis of amino acids	AT2G21170.1 Symbols: triosephosphate isomerase	gluconeogenesis; glycolytic process; triglyceride mobilization; reductive pentose-phosphate cycle; glycerol catabolic process; multicellular organism reproduction; glyceraldehyde-3-phosphate biosynthetic process;
				*Glyma.19G186400*	Phosphatidylinositol signaling system		
				*Glyma.19G186600*	Ribosome biogenesis in eukaryotes	AT3G21540.1 Symbols: transducin family protein / WD-40 repeat family protein	maturation of SSU-rRNA;

KEGG enrichment analysis was performed on QTLs qWSPC-2, qWSPC-8, and qWSPC-20, each exhibiting a genetic contribution greater than 10% within a single evaluation year. Analysis revealed that within the qWSPC-2 locus, a cluster of genes were significantly associated with pathways involved in protein export, Biosynthesis of amino acids, and inositol phosphate metabolism (Figure S1, Table S4). Within the boundaries of qWSPC-8, genes showed enrichment in biological processes including protein binding, activation of protein kinases, and modulation through protein phosphorylation (Table S5). Furthermore, the qWSPC-20 locus harbored genes enriched across 25 distinct pathways, noteworthy among them were the mRNA surveillance pathway, Biosynthesis of amino acids, and the proteasome system. Notably, the gene Glyma.20G213900 within this region might play a pivotal role in the processing of Protein processing in endoplasmic reticulum. (Figure S2, Table S6).

## Discussion

Soybean was domesticated approximately 5000 years ago^[4]^, and the long-term process of domestication has been accompanied by great changes in traits such as seed size, color, dormancy, flowering period, and plant structure [23]. For example, wild soybeans have characteristics such as thin and sprawling stems, small pods, black seed color, uneven maturation of seeds, pod shattering tendencies, and impermeability of seed coats [24]. Meanwhile, regional soybean landraces have the characteristics of smaller plants, pod shattering tendencies, large seeds, various seed colors, and enhanced seed coat impermeability, while developed cultivars have short and stout stems, large seeds that are mostly yellow in color, high seed oil content, and high yield [25, 26]. In addition to morphological changes, the levels of lipids, proteins, and other nutrients in soybean seeds also changed significantly during domestication. Wild soybeans have a lower oil content, while cultivated soybeans have a higher oil content [27]; thus, oil content is considered a trait of soybean domestication [28]. It is not clear whether WSP content has also improved through domestication, and reports on WSP content in wild soybeans are scarce. Hence, in this study, WSP levels were measured in 30 wild soybean germplasm resources and 30 cultivated soybean varieties, revealing that the WSP content was significantly greater in cultivated varieties than in wild soybeans. This finding implies that WSP content could also be a trait of domestication.

Soybeans originated in China and different soybean ecotypes have developed over time. Fully utilizing the rich variation in different ecotype germplasm resources is highly important for broadening the genetic base of soybean varieties. Wild and semiwild soybeans, as ancestors of cultivated soybeans, old vast potential for genetic variation [29, 30]. While wild soybeans are known as high-protein-content ancestors and contain genes responsible for high-protein content, studies exploring the genetic background traits related to WSP content in wild soybeans are scarce. In this study, a population of recombinant inbred lines (RILs) constructed from crosses between wild and cultivated soybeans was used to measure the WSP content, and the qWSPC15 additive effect QTL was detected, indicating that the paternal wild soybean contributed to the increase in genes, partially explaining the overdominance phenomenon in the RIL phenotype. These results suggest that despite the overall low WSP content, wild soybeans still possess elite alleles that can increase WSP content. The identification of this locus provides a theoretical basis for further increasing the WSP content in cultivated soybeans using wild soybean genetic resources. However, since qWSPC15 was detected only in a single year, its genetic and environmental stability requires further investigation.

To date, few studies have identified QTLs related to soybean WSP content. In this study, 8 QTLs for WSP content were identified using an RIL population in the field environments of 2020 and 2022, with 6 QTLs overlapping or closely related to those reported in previous satudies. qWSPC-2 overlaps with a WSP QTL detected by Chen et al. [14], qSPC-2-2. qWSPC-8 has been identified as a major regulatory locus in various populations and environments [11–13, 31]. qWSPC-11 matches the region identified by Shen et al. [32]. qWSPC-15 is close to the loci identified by Zhang et al. [33]. and Chen et al. [14]. The qWSPC-19 locus found in this study also overlaps with regions identified by Zhang et al. [3, 12]. in two successive years, indicating that qWSPC-19 is a major-effect and stable locus. Furthermore, qWSPC-20 is closely related to a locus identified by Zhang et al. [12]. These loci have been repeatedly detected in various environments and genetic backgrounds, suggesting that they could be stable loci regulating soybean WSP content. This study also revealed two new QTLs, qWSPC-7 and qWSPC-14, which were not reported in previous studies but may be related to the specific genetic backgrounds of the materials used.

The WSP content is an important component of the overall soybean protein content, and the nutritional components of soybeans primarily accumulate during seed formation [34]. The protein content in soybean seeds tends to increase slowly first and then rapidly [35]. A search of the SoyBase database revealed that five genes exhibit specific expression patterns at different seed development stages, suggesting their involvement in the accumulation of WSP in soybean seeds. Among them, Glyma.19G185700 and Glyma.19G186000 tended to increase in expression from day 10 to day 17 of seed development, peaking on day 21, which could indicate progressive protein accumulation in soybean seeds. The protein encoded by Glyma.19G186000 is involved in amino acid biosynthesis, one of the basic metabolic functions in plants, and plays an important role in protein synthesis and accumulation. In wheat, research has shown that the major regulatory gene TaGCN2 of amino acid biosynthesis-related enzymes affects the accumulation of free asparagine, reducing the risk of acrylamide formation in wheat products [36]. Similarly, the aspartic acid family (Lys, Thr, Met, and Ile) is crucial for amino acid synthesis and thus plays a significant role in protein accumulation and synthesis [13]. Therefore, Glyma.19G185700 and Glyma.19G186000 are hypothesized to regulate the accumulation of WSP in soybean seeds by controlling pathways such as amino acid biosynthesis.

## Conclusion

This study used a population of 180 F10 recombinant inbred lines (RILs) constructed from the cultivated variety Ji Dou 12 and the wild variety Ye 9 to measure the WSP content in parent and RIL populations in field environments in 2020 and 2022. Based on a high-density genetic map, 8 QTLs associated with WSP content were detected, with two of those loci being reported for the first time. Through KEGG analysis, 5 genes related to WSP were identified within the qWSPC-19 locus, and further expression analysis revealed that the Glyma.19G185700 and Glyma.19G186000 expression levels were significantly greater in Ye 9 than in Ji Dou 12. These two genes, as key candidate genes for qWSPC-19, are likely to function in regulating the WSP content in soybeans.

### Electronic supplementary material

Below is the link to the electronic supplementary material.

Supplementary Material 1

Supplementary Material 2

## Data Availability

The datasets supporting the conclusions of this article are included within the article.Candidate gene predictions supporting the results of this study have been stored in the Phytozome database and the Soybase database. The main login links are ( https : / / phytozome.org ) and ( https://www.Soybase.Org) .
