# Supplementary material for: Identification of QTLs and candidate genes for water-soluble protein content in soybean seeds
Source: BMC Genomics. 2024 Aug 13;25:783. doi: 10.1186/s12864-024-10563-0 (PMC11320831; doi:10.1186/s12864-024-10563-0)
Supplement: Supplementary file 1 — Supplementary Material 1 [file 12864_2024_10563_MOESM1_ESM.pdf]

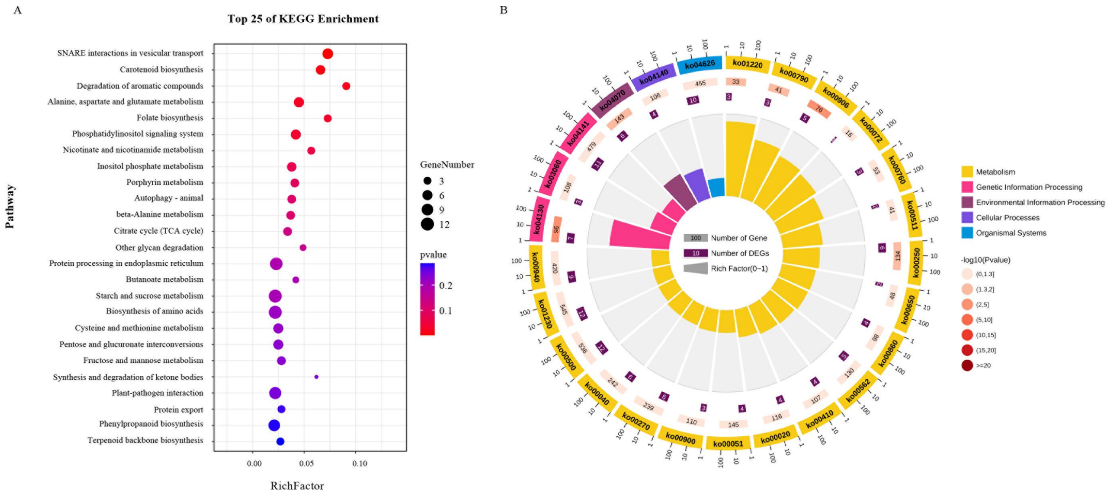

Fig.S1 KEGG analysis of *qWSPC-2* positioning interval genes.

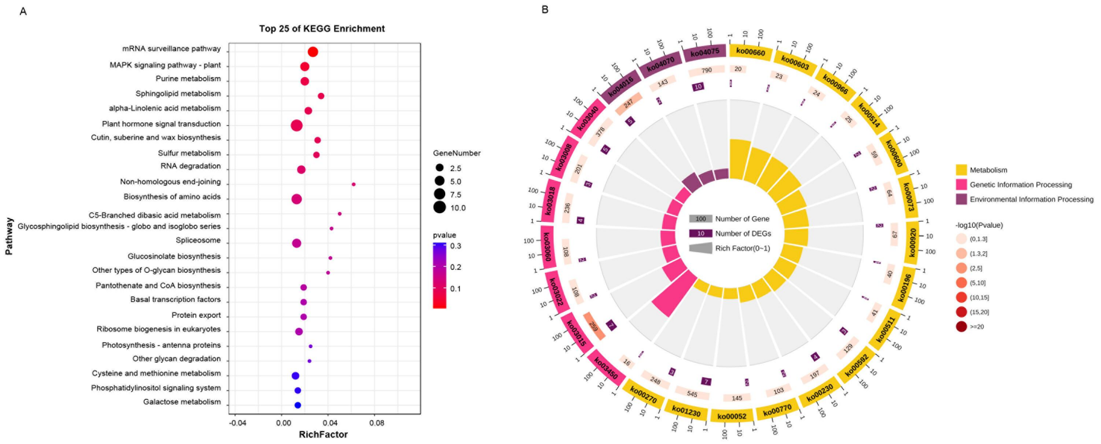

Fig.S2 KEGG analysis of *qWSPC-20* positioning interval genes.
